# GnRH agonist-only trigger, compared to dual trigger, reduces oocyte retrieval rate in high responders without affecting cumulative live birth rate

**DOI:** 10.3389/fendo.2024.1461317

**Published:** 2024-08-20

**Authors:** Yu Wang, Yu-Chiao Yi, Hwa-Fen Guu, Ya-Fang Chen, Hsiao-Fan Kung, Jui-Chun Chang, Li-Yu Chen, Shih-Ting Chuan, Ming-Jer Chen

**Affiliations:** ^1^ Division of Reproductive Endocrinology and Infertility, Department of Obstetrics, Gynecology and Women’s Health, Taichung Veterans General Hospital, Taichung, Taiwan; ^2^ Department of Obstetrics and Gynecology, School of Medicine, National Yang Ming Chiao Tung University, Hsinchu, Taiwan; ^3^ Division of Infertility, Lee Women’s Hospital, Taichung, Taiwan

**Keywords:** *in vitro* fertilization (IVF), dual trigger, GnRH agonist, live birth, oocyte retrieval rate

## Abstract

**Introduction:**

This study compared, in high responders undergoing IVF treatment, GnRH agonist-only trigger and dual trigger on oocyte retrieval rate and cumulative live birth rate (LBR). The aim was to determine if the GnRH agonist-only triggers had provided outcomes comparable to dual trigger, while minimizing the risk of ovarian hyperstimulation syndrome (OHSS).

**Materials and methods:**

A retrospective, matched case-control study was conducted at Taichung Veterans General Hospital, Taiwan, including women who underwent IVF/ICSI between January 1, 2014, and December 31, 2022. Inclusion criteria were: GnRH antagonist protocol and estrogen level >3,000 pg/ml on trigger day. Exclusion criteria were: immune/metabolic diseases, donated oocytes, and mixed stimulation cycles. Propensity score matching was applied to balance age, AMH level, and oocyte number between the GnRH agonist-only and dual trigger groups. Outcomes were analyzed for patients who had complete treatment cycles, focusing on oocyte retrieval rate and cumulative LBR.

**Results:**

We analyzed 116 cycles in the agonist-only group, and 232 cycles in the dual trigger group. No inter-group difference was found in their age, BMI, and AMH levels. The dual trigger group had a higher oocyte retrieval rate (93% vs. 80%; p <0.05), while fertilization rates, blastocyst formation rates, and cumulative LBR were comparable. Notably, no OHSS cases had been reported in the GnRH agonist-only group, compared with 7 cases in the dual trigger group.

**Conclusion:**

GnRH agonist-only triggers resulted in a lower oocyte retrieval rate compared to dual triggers but did not significantly affect cumulative LBR in high responders. This approach effectively reduces OHSS risk without compromising pregnancy outcomes, making it a preferable option in freeze-all strategies, despite a longer oocyte pick-up duration and a medium cost. GnRH agonist-only trigger, however, may not be suitable for fresh embryo transfers or patients with low serum LH levels on trigger day.

## 1 Introduction

Assisted reproductive technology (ART) has significantly transformed the landscape of infertility treatment, enabling countless couples to realize their dream of parenthood. Central to this success is the precise timing of oocyte pick-up before ovulation (1).

In the natural cycle, the midcycle surge represents a noticeable hourly rise in the amplitude of the follicle-stimulating hormone (FSH) and luteinizing hormone (LH) pulses for approximately 48 hours. However, the short half-life of LH makes it impractical for use as an ovulation trigger in ART cycles (2). To enhance the outcomes of *in vitro* fertilization (IVF), a range of protocols have been developed and tested over time, with a focus on the use of gonadotropin-releasing hormone (GnRH) agonist and human chorionic gonadotropin (hCG) triggers.

Ovarian hyperstimulation syndrome (OHSS) commonly occurs following ovarian stimulation with gonadotropins for ART. The defining characteristic of OHSS is increased capillary permeability, which leads to ascites and pleural effusion. Vascular endothelial growth factor (VEGF) is the primary molecule responsible for this increased vascular permeability (3). The number of luteinized granulosa cells determines the incidence and severity of the syndrome (4). Consequently, patients who are younger and have a better ovarian reserve are at a higher risk of developing OHSS (5). Mild OHSS is typically self-limiting. However, severe OHSS can lead to significant complications such as massive ascites, pleural effusion, renal failure, oliguria, hypotension, and thrombosis. To prevent OHSS, the primary strategy is to limit the number of developing follicles. Secondary measures include the use of certain medications, such as dopamine agonists and angiotensin-converting enzyme inhibitors (6, 7). Nonetheless, the most effective prevention is to lower or avoid the use of HCG during the trigger phase (8).

As a trigger for the final maturation of oocytes, hCG, like LH, has been in use for >50 years (9, 10). It induces ovulation approximately 38-40 hours post trigger, mimicking the natural ovulation time and is long regarded as the gold standard (2). However, its extended biological effect increases the risk of OHSS, particularly in high responders. In contrast, the use of the GnRH agonist trigger has demonstrated improvements in both oocyte quality and quantity while reducing OHSS risk due to a shorter duration of LH elevation (11).

GnRH agonist-only trigger leads to luteolysis and corpus luteum insufficiency (12). Recent advancements have introduced the concept of a dual trigger, combining GnRH agonist and a reduced hCG dosage. This strategy, in GnRH antagonist cycles, aims to merge the benefits of GnRH agonist trigger with the luteal support provided by hCG, thereby mitigating premature luteinization risks and enhancing pregnancy outcomes (13). Moreover, it significantly enhances oocyte maturation, particularly in individuals with historically low rates of mature oocyte retrieval (14).

Despite the potential advantages of GnRH agonist and dual triggers, conflicting evidence exists on their effectiveness in high responders. Some studies suggest improved pregnancy outcomes with the GnRH agonist trigger, while others indicate poorer pregnancy rates compared to hCG triggers (15, 16). Similarly, outcomes with the dual trigger vary, with some studies reporting enhancements and others no improvement compared to the GnRH agonist trigger. These discrepancies may be due to differences in patient profiles, study methodology, and trigger protocol, necessitating further research for their clarification.

Considering the growing adoption of freeze-all strategy, the necessity of dual triggering is questioned. The main drawback of GnRH agonist-only trigger lies in luteal-phase insufficiency, markedly impacting implantation and pregnancy rates in the fresh embryo transfer (ET) (17). However, this downside can be easily overcome by a freeze-all strategy.

In this study, we aimed to compare outcomes between GnRH agonist-only trigger and dual trigger in high responders using a GnRH antagonist protocol and freeze-all strategy. We hope to refine trigger methods for high responders in GnRH antagonist cycles.

## 2 Materials and methods

### 2.1 Subjects

This retrospective, matched case-control study was conducted at Taichung Veterans General Hospital, Taiwan. We enrolled women who had received IVF and/or ICSI during the period between January 1, 2014, and December 31, 2022. Inclusion criteria were as follows: (1) patients received GnRH antagonist protocol, (2) induction duration > 5 days, (3) estrogen level > 3000 pg/ml on the trigger day. Exclusion criteria were as follows: (1) patients with immune diseases or metabolic diseases, (2) patients receiving donated oocytes, (3) patients with embryos transferred from mixed stimulation cycles.

Propensity score matching was applied to select matched subjects with balanced age, anti-Mullerian hormone (AMH) level, and retrieval oocyte number, and at ratio of 1:2 between GnRH agonist-only trigger and dual trigger groups.

We analyzed patients who had completed their treatment cycles, either as the result of achieving a live birth or, ultimately, failing after transferring all of their embryos.

### 2.2 Controlled ovarian stimulation protocol

Patients received the GnRH antagonist cetrorelix acetate (Cetrotide, 0.25 mg/d SC; Merck Serono, Germany) starting on a flexible plan from stimulation days 5 to 7 with ultrasound monitoring 5 days after the onset of controlled ovarian hyperstimulation with gonadotropins.

The types and dosages of gonadotropin administration were individualized for each participant according to her age, body mass index, AMH, FSH/LH level, antral follicle counts on cycle days 2 to 3 and previous responses to ovarian stimulation. Dosages were adjusted according to the ovarian response as monitored by vaginal ultrasound folliculometry and serum estrogen (E2) level.

### 2.3 Triggering and luteal support protocol

When two or more follicles reached a mean diameter of 18 mm, an ovarian trigger was arranged. For the GnRH agonist-only trigger, patients were triggered with 0.2 mg Decapeptyl (Ferring Co., Germany). For dual trigger, patients were triggered with the same regimen of GnRH agonist and 250 mcg of recombinant hCG (Ovidrel; Merck Serono, Germany). Oocyte retrieval was performed 35 to 36 hours later. Progesterone 25mg/amp (Astar Co., Taiwan), 1 to 2 amp/day, was injected intramuscularly starting from the night of oocyte retrieval and continued or shifted to topical progesterone (8% Crinone; Merck-Serono, Germany) 90mg/day on the day of embryo transfer. The procedure was maintained until the pregnant patients had reached 8 complete weeks of gestation for luteal support (LS).

For the frozen-thaw cycle (FET), patients received an artificial hormone replacement regimen (Estradiol valerate 2 mg, Synmosa Co., Taiwan) with a step-up dose from 4 to 8mg/day for 5 days, to 6 to 12mg/day for 5 days, followed by twice daily doses of vaginal progesterone (8% Crinone; Merck Serono, Germany) at 90mg, plus estradiol valerate 12mg once the endometrial thickness had exceeded 8 mm on ultrasound images. Finally, dosage was maintained till the patient had reached 10 complete weeks of gestation for LS.

In addition, 0.1 mg Decapeptyl (Ferring Co., Germany) was also administered on day 6 after the start of luteal support. Embryo transfers (ET) were carried out on day 2, day 3, or day 5 of culture.

### 2.4 Outcome measures

The live birth rate (LBR) was defined as the percentage resulting in live newborns. The cumulative LBR per retrieval cycle was defined as the percentage of at least one live newborn from that retrieval cycle. The above results were followed up until 30 September 2023.

### 2.5 Statistical analyses

We applied the greedy nearest neighbor matching to calculate the propensity scores matched in baseline parameters at a ratio of 1:2 for Group 1 and 2. Categorical items were described using percentage and frequency, whereas continuous characteristics were assessed using descriptive statistics (number of subjects, mean, standard deviation). Parameters were compared using the two-sample t-test for continuous variables, and the comparison between categorical variables was conducted with the chi-square test. Significance was set at p <0.05. All analyses were performed on the SPSS-PC ver. 22.0 (SAS Institute Inc, Cary, NC, USA).

### 2.6 Editorial board members and editors

The study protocol was approved by the Ethics Committee of the Taichung Veterans General Hospital, No. CE24173B on 2024/04/05, and adhered to the relevant ethical guidelines. The Institutional Review Board of Taichung Veterans Hospital approved to waive the documentation of informed consent due to this research presented no more than minimal risk of harm to subjects which involves only data review.

## 3 Results

Our analysis was conducted on a total of 3822 cycles, including 116 cycles in the agonist-only trigger group and 232 cycles in the dual trigger group.

**Table 1** shows no significant difference between the two groups regarding age, BMI, and AMH levels. In terms of stimulation cycle characteristics, the FSH dose was higher in the dual trigger cycles. Although the predicted number of oocytes by number of follicles with diameter ≥ 14 mm was higher in GnRH agonist-only trigger cycles, the retrieved oocyte count remained similar across both groups following case-matching. **Table 2** shows that the oocyte retrieval rate appeared higher in the dual trigger group. There was no inter-group difference in fertilization rate, blastocyst formation rate and good embryo rates in both day 3 and day 5. We noted no OHSS had been documented in the GnRH agonist-only trigger group, whereas 7 patients experienced OHSS in the dual trigger group.

**Table 1:** Baseline demographics and cycle characteristics of patients with agonist-only versus dual trigger.

Trigger protocol	GnRH agonist only (n= 116)	hCG + GnRH agonist (n=232)	P value
Age (years)	34.5± 4.1	34.3 ± 2.8	0.635
BMI (kg/m^2^)	22.6± 3.7	22.3 ± 3.7	0.471
AMH (ng/ml)	6.14 ± 3.0	6.13 ± 3.65	0.139
Total FSH dosage (IU)	2457.8 ± 841.9	2740.4 ± 951.4	0.040*
Total LH dosage (IU)	725.4± 532.4	794.9± 725.4	0.240
Induction duration (days)	10.9 ± 0.99	10.43 ± 1.21	0.372
Endometrium thickness (mm)	11.8± 2.4	11.7 ± 3.1	0.909
No. of ≥ 14mm follicle	18.4 ± 8.10	15.2 ± 6.0	<0.001**
Estradiol on day of hCG administration (pg/ml)	5622.1 ± 1695.5	4931.6 ± 1888.7	0.002*

*p < 0.05.

**p<0.01.

**Table 2:** Embryology results of patients with agonist-only versus dual trigger.

Trigger protocol	GnRH agonist-only (n= 116)	hCG + GnRH agonist (n=232)	P value
No. of oocytes retrieved	24.0 ± 7.7	23.7 ± 8.2	0.383
Fertilization oocyte numbers	17.0 ± 7.1	16.3 ± 7.1	0.424
Oocytes retrieval rate (retrieved oocytes/number of follicles with diameter > 14 mm)	80%	93%	<0.001**
Fertilization rate	71%	70%	0.426
Good embryo ^a^ numbers (at day3)	5.3 ± 4.0	6.0 ± 4.5	0.179
Rate of good embryos ^a^ (at day3)	29% ± 16%	34% ± 21%	0.268
Blastocyte formation rate	53% ± 22%	55% ± 24%	0.646
Good blastocyst ^b^ numbers	7.0 ± 5.0	6.4 ± 5.1	0.623
Rate of good blastocyst ^b^	39% ± 22%	39% ± 23%	0.781
Ovarian hyperstimulation syndrome (OHSS)	0 cycle	7 cycles	

** p<0.01.

a Cleavage stage embryo was defined as good quality embryos if they were composed of at least seven to eight cell grade 1 or 2 on day 3, according to the Veeck classification system.

b Grade according to Gardner classification system of “3BA” or greater was defined as good quality blastocyst.

As for frozen embryo transfer, both groups utilized 100% artificial cycles. Information on the number of day 2 or day 3 embryos frozen in both groups is as follows: In the dual trigger group, 4 patients froze day 2 or day 3 embryos: Patient 1 froze 2 day 2 embryos. Patient 2 froze 6 day 2 embryos. Patient 3 froze 6 day 3 embryos. Patient 4 froze 6 day 3 embryos and 4 day 5 embryos. In the GnRH-only trigger group, only 1 patient froze 4 day 2 embryos. Additionally, no patients in the GnRH-only trigger group received fresh embryo transfer (ET), while 59 patients (25.1%) in the dual trigger group received fresh ET.

**Figure 1:**
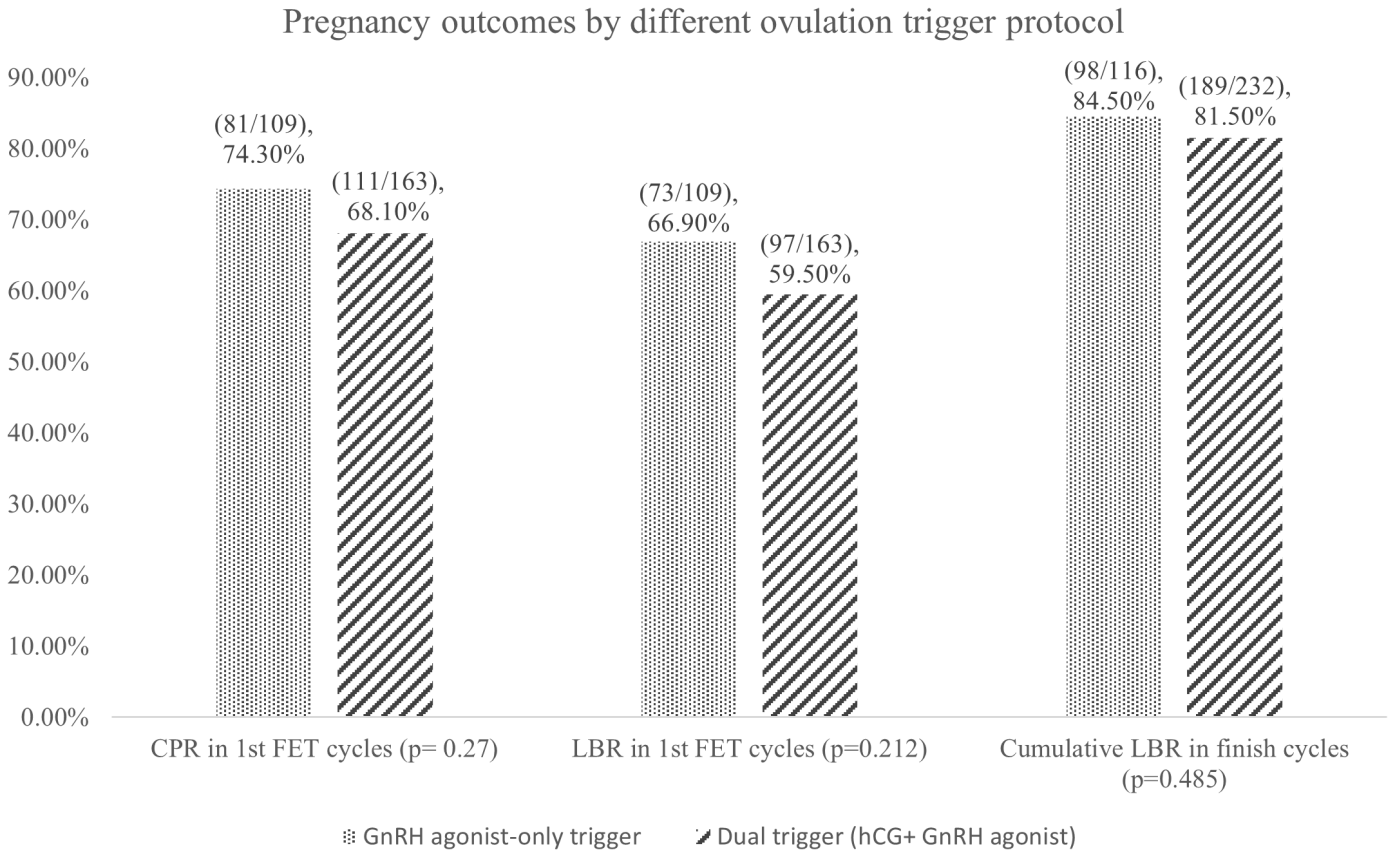
Pregnancy outcomes by different ovulation trigger protocol. FET, frozen embryo transfer; CPR, clinical pregnancy rate; LBR, live birth rate.

**Figure 1** shows no statistically significant difference between the two groups regarding clinical pregnancy rate (CPR), LBR, and cumulative LBR. Particularly remarkable was a >80% cumulative LBR in both groups. **Table 3** showed the total number of ET cycles.

**Table 3:** Number of embryo transfer cycle in both groups.

Number of ET cycle	<=1	2	3	4	5	6
Patient number in dual trigger group (n=232)	147 (63.4%)	54 (23.3%)	24 (10.3%)	4 (1.7%)	2 (0.86%)	1 (0.43%)
Patient number in GnRH-only trigger group (n=116)	69 (59.5%)	23 (19.8%)	13 (11.2%)	6 (5.2%)	2 (1.7%)	3 (2.6%)

The distribution of embryo transfer numbers for different trigger groups and the average numbers of transferred embryos and good embryos are shown in **Tables 4**, **5**. The data indicates that in single embryo transfer cycles, the dual trigger groups have a higher number of embryo transferred.

**Table 4:** Comparison of embryo transfer cycle numbers and average transfer numbers between GnRH agonist-only trigger and hCG+GnRH agonist trigger groups.

	GnRH agonist-only trigger	hCG+ GnRH agonist	P value
Day 2 embryo transfer cycle numbers	0	2 (0.62%)	
Day 3 embryo transfer cycle numbers	0	5 (1.55%)	
Day 5 embryo transfer cycle numbers	198 (100%)	315 (97.83%)	
Average transfer number of Day 2 embryo		3	
Average transfer number of Day 3 embryo		3	
Average transfer number of Day 3 good embryo ^a^		1.4	
Average transfer number of Day 5 embryo	1.58	1.75	<0.001**
Average transfer number of Day 5 good embryo ^b^	1.5	1.64	0.006**

**p<0.01.

^a^Cleavage stage embryo was defined as good quality embryos if they were composed of at least seven to eight cell grade 1 or 2 on day 3, according to the Veeck classification system.

^b^Grade according to Gardner classification system of “3BA” or greater was defined as good quality blastocyst.

**Table 5:** Distribution of embryo transfer numbers of different trigger groups and days of embryo development.

Embryo transfer number	1	2	3	4	>5
GnRH agonist-only trigger group Day 5 cycle numbers	87 (43.94%)	108 (54.55%)	3 (1.52%)	0	0
hCG+ GnRH agonist trigger group Day 2 cycle numbers	0	1 (50%)	0	1 (50%)	0
hCG+ GnRH agonist trigger group Day 3 cycle numbers	0	1 (20%)	3 (60%)	1 (20%)	0
hCG+ GnRH agonist trigger group Day 5 cycle numbers	93 (29.52%)	209 (66.35%)	12 (3.81%)	1 (0.32%)	0

## 4 Discussion

In IVF, LH plays a crucial role in activating oocyte maturation and resuming meiosis before oocyte retrieval. Various methods, such as using hCG and GnRH agonists, mimic the physiological LH surge necessary for IVF treatment. The historical gold standard is hCG, owing to its similar alpha subunit to LH (23, 24). However, its extended half-life is a disadvantage, since it contributes to the higher risk of OHSS, particularly in patients with higher AMH levels and PCOS (25, 26). Numerous strategies, like minimizing hCG dosage, and FET, have been employed to mitigate the OHSS risk. The most effective approach appears to be replacing hCG trigger with a GnRH agonist, which offers several advantages over hCG. GnRH agonists not only mimic LH but also provide FSH, resembling a more physiological cycle and can potentially promote nuclear maturation (27, 28). But the shorter LH surge induced by GnRH agonist may lead to insufficient luteal function, resulting in low ongoing pregnancy rates and greater early pregnancy loss (15, 29, 30). Studies also indicated more failures in collecting mature oocytes following GnRH agonist trigger (31). The results of previous studies are shown in **Table 6**.

**Table 6:** Comparison of published studies: GnRH agonist-only trigger versus dual trigger (hCG + GnRH agonist) and pregnancy outcomes.

Study design	Number of patients (GnRH agonist-only trigger/dual trigger)	Treatment protocols and IVF characteristics	GnRH agonist-only trigger outcomes	Dual trigger outcomes	OHSS rate (GnRH agonist-only trigger/dual trigger)	Authors/year
Retrospective cohort study	108/66	Antagonist protocol/high responders	Number of oocytes retrieved: 16.5/pregnancy outcomes not available	Total number oocytes retrieved 17.5 /pregnancy outcomes not available	0/6%	O’Neil et al./2016 (18)
Retrospective cohort study	232/59	Antagonist protocol/oocyte donation cycles	Number of oocytes retrieved: 14/pregnancy outcomes not available	Number of oocytes retrieved: 13.5/pregnancy outcomes not available	0/8.5%	Jones et al./2019 (19)
Retrospective cohort study	959/50	Antagonist protocol/oocyte cryopreservation cycles	Number of oocytes retrieved: 18.3/pregnancy outcomes not available	Number of oocytes retrieved: 18.24/pregnancy outcomes not available	0/0	Maslow et al./2020 (20)
Retrospective cohort study	577/403	Antagonist protocol	Number of oocytes retrieved: 22.93/LBR 54.22%/cLBR 74.35%	Number of oocytes retrieved: 22.41/LBR 54.20%/cLBR 74.35%	0/1.49%	Y. He et al./2022 (21)
Randomized controlled trial	164/168	Antagonist protocol/advanced-age women (≥ 35 years)	Retrieval rate 84.1%/Number of oocytes retrieved: 3.81/LBR 14.1%	Retrieval rate 87.9%/Number of oocytes retrieved: 4.08/LBR 32.6%	No data	Zhou et al./2022 (22)

OHSS, Ovarian hyperstimulation syndrome; LBR, Live birth rate; cLBR, cumulative live birth rate.

The dual trigger approach offers the advantage of combining both GnRH agonist and hCG, thereby reducing the OHSS risk by minimizing hCG dosage while it can also induce endogenous FSH and LH surges. Studies have shown that in poor responders, dual trigger could lead to an increase in the number of mature oocytes, CPR, and LBR (32, 33). Several questions remain unanswered regarding the use of dual trigger. For instance, should dual triggers be applied to those patients with a higher risk of OHSS? Is dual trigger superior to the GnRH agonist alone? And does it impact the oocyte retrieval rate? Only a limited studies have so far directly compared GnRH agonists with dual triggers. Two randomized controlled trials (RCTs) compared hCG and dual trigger (34, 35). Hass et al. concluded that dual trigger can result in higher CPR and LBR per transfer compared to hCG alone (34). Similarly, Maged et al., in a study involving poor ovarian responders undergoing ICSI and a GnRH-antagonist protocol, found that dual trigger is associated with higher chemical pregnancy rates compared to hCG alone (35). These findings highlight the potential benefits of the dual trigger approach, particularly in certain patient populations.

The use of GnRH agonist alone as a trigger restricted in clinical practice due to reports on its poorer pregnancy outcomes, particularly among patients who may exhibit insufficient LH production under GnRH agonist trigger, affecting final oocyte maturation (36). O’Neill et al. suggested that dual trigger protocols may enhance the number of retrieved oocytes and improve their maturation compared to GnRH agonist-only triggers (18). In a randomized control study including patients of relatively advanced ages, GnRH agonist-only triggers have a lower oocyte retrieval rate and lower LBR (22). However, conflicting findings exist, with some studies indicating no significant differences in outcomes between GnRH agonist-only triggers and dual triggers. For instance, Jones et al. found that both GnRH agonist and dual trigger groups present a higher number of mature oocytes compared to hCG-only trigger (19). Similarly, Maslow et al. reported no difference in oocyte maturation between patients given GnRH agonist-only triggers and dual triggers, regardless of estrogen levels on the trigger day (20). He et al, in a retrospective study, reported that among patients retrieving over 15 oocytes with a freeze-all strategy, no extra benefit was found in using hCG compared to GnRH agonist-only triggers (21). These studies also reported no discrepancies in the following: rates of oocyte maturation, fertilization, top-quality day 3 embryos, cumulative LBR, and neonatal outcomes. Regarding fresh embryo transfer cycles, GnRH agonist triggers are associated with poorer pregnancy outcomes. However, in freeze-all cycles, the outcomes of FET appear excellent (37, 38). In our study, we similarly noticed no difference in pregnancy rates, in terms of 1^st^ frozen ET pregnancy rate or cumulative LBR.

In our study, we observed a difference in oocyte retrieval rates between the two groups, with a rate of 93% in the dual trigger group and 80% in the agonist-only trigger group. This finding is consistent with a previous randomized controlled study conducted by Zhou et al. (22). It is our standard procedure to aspirate all follicles larger than 10mm and perform a maximum of 6 flushes if no oocytes have been detected under the microscope during an oocyte retrieval. Our embryologist typically would examine the fluid retrieved during oocyte pick-up in real-time to confirm successful oocyte retrieval. According to our own real-world experiences, we encountered more frequent difficulties in oocyte retrieval in agonist only trigger cycles. The use of GnRH agonist-only triggers may lead to a longer procedure time and consequently increase the consumption and cost of the medium.

Previous studies have shown that a dual trigger may result in a better good embryo rate (18, 22); however, we did not observe this finding in our research. In our study, we hypothesize that the GnRH-agonist-only trigger may result in a lower oocyte retrieval rate. Although the rate of good-quality embryos is similar between the GnRH agonist-only trigger and dual trigger groups, the total number of good embryos may be lower due to the reduced number of retrieved oocytes in the former group. This reduction could be attributed to the retrieval process selectively capturing more mature oocytes or those with better potential to develop into good embryos. A 2016 study also observed that adding a bolus of hCG significantly increased the oocyte retrieval rate but did not affect oocyte maturity or fertilization rate (39). In our clinical experience, we have often noticed that an agonist-only trigger is associated with a lower retrieval rate. This may be due to the lack of hCG to induce mucification of granulosa cells (40). We agree that a dual trigger can improve the oocyte retrieval process. However, considering the risk of OHSS and the cumulative live birth rate, it may be beneficial for some patients to accept a slightly lower oocyte retrieval rate in exchange for a reduced risk of OHSS without compromising the cumulative live birth rate.

The limitations of this study are as follows: first, it is not a randomized study. Additionally, the sample size is relatively small. While all oocyte pick-up procedures were performed at our hospital using the same equipment, they were conducted by different doctors, which could introduce variability in the procedure. Furthermore, the retrospective nature of the study may have inherent biases, and cannot completely eliminate all potential confounding factors.

In conclusion, the use of GnRH agonist-only trigger results in (a) similar parameters of embryonic development and (b) comparable cumulative pregnancy rates, as compared to dual trigger in patients with estrogen levels exceeding 3000 pg/ml on the trigger day. For these patients, GnRH agonist-only trigger is preferable due to no difference in the final reproductive outcomes, and the nearly risk-free on OHSS compared to dual triggers. However, it should be noted that the oocyte retrieval rate was significantly lower in the GnRH agonist-only trigger group and that potentially would lead to an extended duration of oocyte pick-up procedure. Also, it may not be a good choice for patients who wish to undergo fresh ET or have low serum LH levels on the day of trigger, since a lower LH level is associated with reduced ongoing pregnancy rates, LBR, and higher miscarriage rates with the GnRH agonist-only trigger (41). In summary, while the use of GnRH agonist alone as the trigger agent showed comparable pregnancy rates and may mitigate the risk of OHSS, it is important to consider the lower oocyte retrieval rate and potential overload in medium cost and procedure time associated with this approach.

## Data Availability

The original contributions presented in the study are included in the article/supplementary material. Further inquiries can be directed to the corresponding author.
